# Optimizing the methodology for measuring supraclavicular skin temperature using infrared thermography; implications for measuring brown adipose tissue activity in humans

**DOI:** 10.1038/s41598-017-11537-x

**Published:** 2017-09-20

**Authors:** Tahniyah Haq, Justin D. Crane, Sarah Kanji, Elizabeth Gunn, Mark A. Tarnopolsky, Hertzel C. Gerstein, Gregory R. Steinberg, Katherine M. Morrison

**Affiliations:** 1 Department of Pediatrics, McMaster University, Hamilton, L8S 4K1 Canada; 2 Department of Medicine, McMaster University, Hamilton, L8S 4K1 Canada; 3 Department of Biochemistry and Biomedical Sciences, McMaster University, Hamilton, L8S 4K1 Canada

## Abstract

The discovery of brown adipose tissue (BAT) in adults has sparked interest in its role as a therapeutic target in metabolic disorders. Infrared thermography is a promising way to quantify BAT; however, a standardized methodology has not been established. This study aims to establish a standardized and reproducible protocol to measure thermal response to cold in the supraclavicular area using thermographic imaging. In Phase 1, we compared the thermal response to 12 °C cold after acclimation at either 32 °C or room temperature using thermographic imaging. Repeatability of the 32 °C acclimation trial was studied in a second group in Phase 2. Phase 1 included 28 men (mean age 23.9 ± 5.9 y; mean BMI 25.2 ± 3.9 kg/m^2^) and Phase 2 included 14 men (mean age 20.9 ± 2.4 y; mean BMI 23.6 ± 3.1 kg/m^2^). The thermal response was greater after 32 °C than after room temperature acclimation (0.22 ± 0.19 vs 0.13 ± 0.17 °C, *p* = 0.05), was not related to outdoor temperature (r = −0.35, p = 0.07), did not correlate with supraclavicular fat (r = −0.26, p = 0.21) measured with dual-energy x-ray absorptiometry and was repeatable [ICC 0.69 (0.14–0.72)]. Acclimation at 32 °C followed by cold generates a reproducible change in supraclavicular skin temperature measurable by thermal imaging that may be indicative of BAT metabolic activity.

## Introduction

There has been a renewed interest in the potential of brown adipose tissue (BAT) as a therapeutic target to improve metabolic health, especially after confirmation of its presence in adult humans^1–5^. Brown adipocytes are uniquely thermogenic due to their expression of uncoupling protein 1 (UCP1) which is activated by cold, enhances energy expenditure and produces heat^6,7^. Retrospective studies with positron-emission tomography using^18^F-fluorodeoxyglucose and computed tomography (^18^F-FDG PET–CT) indicate that BAT is located primarily in the supraclavicular (SCV) and cervical areas in adult humans^8,9^. Studies using this technique have shown that^18^F-FDG uptake into BAT is greater in females^8–10^, young individuals^8,11^^,^ those with low BMI^2,8,12^ and is enhanced during cooler seasons^1,2,9,13^. Alterations in FDG-uptake are associated with changes in whole body energy expenditure in some but not all studies^5,14,15^. More importantly, activated BAT takes up fatty acids^5,16^ and glucose^5^ from the circulation, thus contributing to their clearance. Fasting blood glucose is lower in those with BAT than those without; and BAT activity is inversely correlated with fasting glucose concentration^7^. There is a lower likelihood of detecting BAT in humans with Type 2 diabetes^9^. Furthermore, 10 days of cold exposure increased insulin sensitivity in adults with Type 2 diabetes^17^, supporting the suggestion that manipulation of BAT may be a potentially important treatment for obesity and type 2 diabetes. This makes it increasingly important to assess the activity and mass of BAT in humans.

Although^18^F-FDG PET–CT is considered the gold standard in the measurement of BAT, its high cost and radiation hazards prohibit widespread use in performing repeated scans or studying pediatric or pregnant populations. Furthermore, since BAT also utilizes both free fatty acids^5^ and triglycerides^16^, changes in^18^F-FDG PET may not be fully reflective of alterations in BAT metabolic activity^18^. Consistent with this concept, mice lacking UCP-1 retain ^18^F-FDG uptake, highlighting a dissociation between this measure and BAT activity^19^. Given these limitations of ^18^F-FDG PET–CT, it is important to develop noninvasive, safe and relatively inexpensive methods to assess BAT metabolic activity.

An alternative methodology for assessing BAT metabolic activity in humans may involve measuring SCV skin temperature using infrared thermography following cold-exposure. Given the proximity of BAT to the skin, a rise in SCV temperature, due to a combination of thermogenesis and increased blood flow, has been suggested to be a measure of BAT activity^20–22^. Consistent with this idea, changes in SCV temperature assessed using infrared thermography have correlated with cold induced^18^F-FDG PET–CT^22^. Furthermore, Crane *et al*. have shown that, in mice, in response to beta-adrenergic stimulation, surface temperature change over the BAT depots detected by infrared thermography is specific for UCP1 mediated thermogenesis^23^. However, in humans, there have been no studies looking at the reproducibility of infrared thermography and the methodology required to facilitate optimal assessment of BAT metabolic activity has not yet been established. Therefore, the current study was undertaken to develop a standardized protocol that will give a maximum and reproducible measure of the thermal response to cold in the SCV area using infrared thermography in adult humans.

## Method

Healthy adult males aged 18 to 39 y were recruited at McMaster University between August 2014 and January 2016. The study was conducted in 2 Phases – (i) Phase 1: determination of the pattern and magnitude of thermal response and (ii) Phase 2: determination of the repeatability of thermal response. The study was approved by the joint Research Ethics Board of McMaster University and Hamilton Health Sciences and all aspects of the study were performed in accordance with that approval. Participants provided informed, signed consent. Exclusion criteria, subject preparation and conditions used in the study can be found in Table 1.

**Table 1:** Criteria and conditions used in the study (Phase 1 and 2).

Characteristics	Criteria and conditions in study
**Exclusion criteria**	
Prescription and over counter medications	Vitamins, supplements
β-blockers, β-adrenergic agonists	None
Habitual tobacco use	None
Habitual excessive alcohol use	None
Plasma glucose	Not recorded (none of the participants were known to have diabetes mellitus)
Chronic disorder	None
Weight change within >5% within 3 months	Not recorded
**Subject preparation**	
Meals 24 hours before visit	Unrestricted diet
Caffeine before visit	No caffeine on the morning of the visit
Fast duration before visit	8–12 hours
Pharmaceuticals	None
Strenuous activity within 48 hours of visit	None
**Study visit conditions**	
Clothing during visit	Light sleeveless T-shirt (CLO 0.06)
Environmental (room) temperature	20–23 °C
Time of day for scan	Between 0630 to 1100 hours
Geographical location	43°15′48′′N 79°55′8′′W
Time of year	Fall, winter, summer and spring (Phase 1) Fall and winter (Phase 2)
Outdoor temperature range	−20.8 to 26.2 °C (Phase 1) −9.2 to 21.6 °C (Phase 2)
**BAT activation / cooling protocol**	
Cooling paradigm	Fixed
Cooling device	Water-perfused cooling blanket (Blanketrol II, Cincinnati Sub-Zero, Sharonville, OH)
Coolant temperature during cooling period	Water temperature in cooling blanket 12 °C Room temperature 20–23 °C
Total duration at cool temperature	60 min
Method used to monitor skin temperature	None
Method used to monitor shivering	Subjective and objective. Surface EMG using a 16-Channel EMG system (Motion Labs Inc.) was done in 5 participants

### Study visits

#### Phase 1 - determination of the pattern and magnitude of thermal response

This Phase required completion of 4 visits scheduled approximately one week apart (to ensure resolution of any residual effect of the cold challenge, described below). Resting energy expenditure (REE) measured at Visit 1 was utilized as a measure to identify participants likely to have BAT. A previous study indicated that individuals with BAT had a 410 ± 293 kcal/d increase in cold-induced resting energy expenditure (REE). REE strongly correlates with BAT activity measured with18FDG PET-CT scans^24^. We therefore chose to use one standard deviation below the mean change in cold-induced REE as a cut off for identifying participants with BAT activity. Therefore, only individuals with an increase in REE of 100 kcal/d following cold exposure were invited for visits 2–4 (thermography visits).

**Figure 1:**
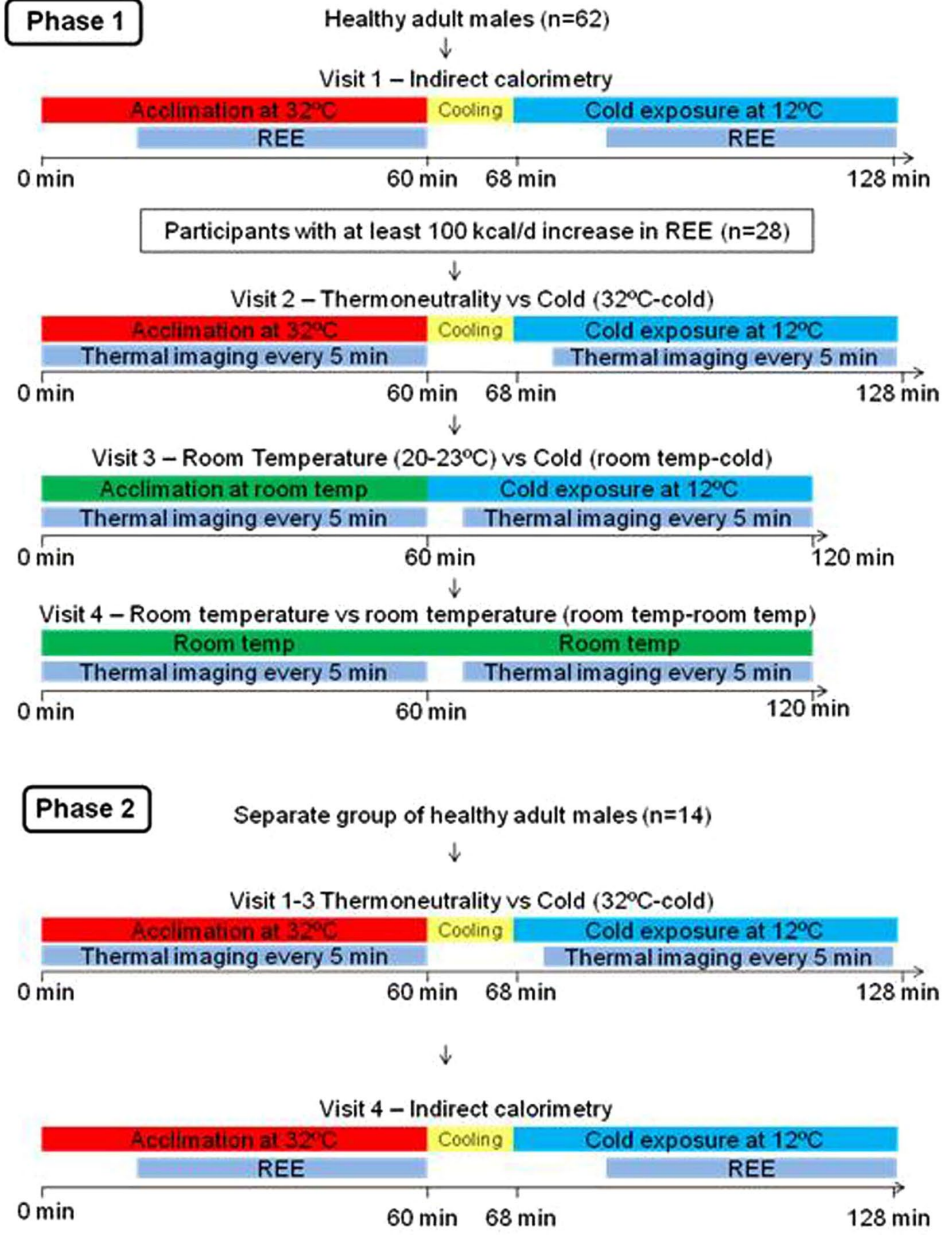
Flow chart of study visits. In Phase 1, 28 out of 62 healthy adult males with 100 kcal/d increase in REE on indirect calorimetry in visit 1 underwent thermography in visits 2–4. In Phase 2, another 14 separate participants underwent 3 trials of thermography repeatability (visits 1–3) irrespective of their change in REE. In each visit, there was an hour of acclimation followed by cold exposure.

In visit 1, steady state REE was recorded by indirect calorimetry over 60 min at 32 °C acclimation followed by 60 min at 12 °C using a water-perfused cooling blanket wrapped around the torso. Visits 2–4 included one hour of acclimation and one hour of controlled exposure and the collection of thermographic images every five min. Visit 2: Acclimation at 32 °C followed by 1 h of 12 °C torso cold exposure (32 °C-cold); Visit 3: acclimation at room temperature (20–23 °C) followed by 1 h of cold exposure (room temp-cold); and Visit 4: acclimation at room temperature followed by 1 h of room temperature (room temp-room temp). In visits where the acclimation temperature was 32 °C, there was an 8-min time period required between acclimation and cooling as the blanket temperature declined from 32 °C to 12 °C. The first image of the 2nd hour was taken 5 min after 12 °C temperature was reached (Fig. 1).

Surface electromyography (EMG) was measured on a separate day in 5 adult male participants with mean BMI of 26.0 ± 3.1 kg/m^2^ who had shown a 100 kcal/d increase in cold induced REE during 1 hour torso cold exposure at 12 °C using a cooling blanket.

#### Phase 2 - determination of the reproducibility of the thermal response

Phase 2 consisted of reproducibility trials which included 3 thermography visits each consisting of an hour of acclimation at 32 °C followed by an hour of cooling at 12 °C (visit 1–3). The 32 °C acclimation trial was chosen in the repeatability trials as it showed a greater and earlier thermal response to cold in Phase 1. Cold-induced REE was measured on another day (visit 4). Each thermography visit was conducted in the same room and approximately 9 days apart. Another 14 new participants were included irrespective of their change in REE to cold to participate in Phase 2 (Fig. 1).

### Study procedures

#### Cooling protocol

Cooling was applied with a cooling blanket (Hyper-Hypothermia unit - Blanketrol II, Cincinatti Sub-Zero, Sharonville, OH) set at 12 °C. The blanket was folded in half lengthwise and placed so it covered the participant’s torso. The temperature was selected based on the literature.

#### Metabolic rate testing

The participant lay on the bed under a canopy ventilation hood connected to a metabolic cart (VmaxTM Encore PFT System, Sensor Medics). Flow sensor and gases were calibrated before each measurement. Steady state was achieved when REE varied by less than 3% for at least 3 min. The average steady state REE over the last 45 min of acclimation was subtracted from that of cold to calculate the increase in cold induced REE and thereby identify participants with and without BAT activity.

#### Thermal imaging

**Figure 2:**
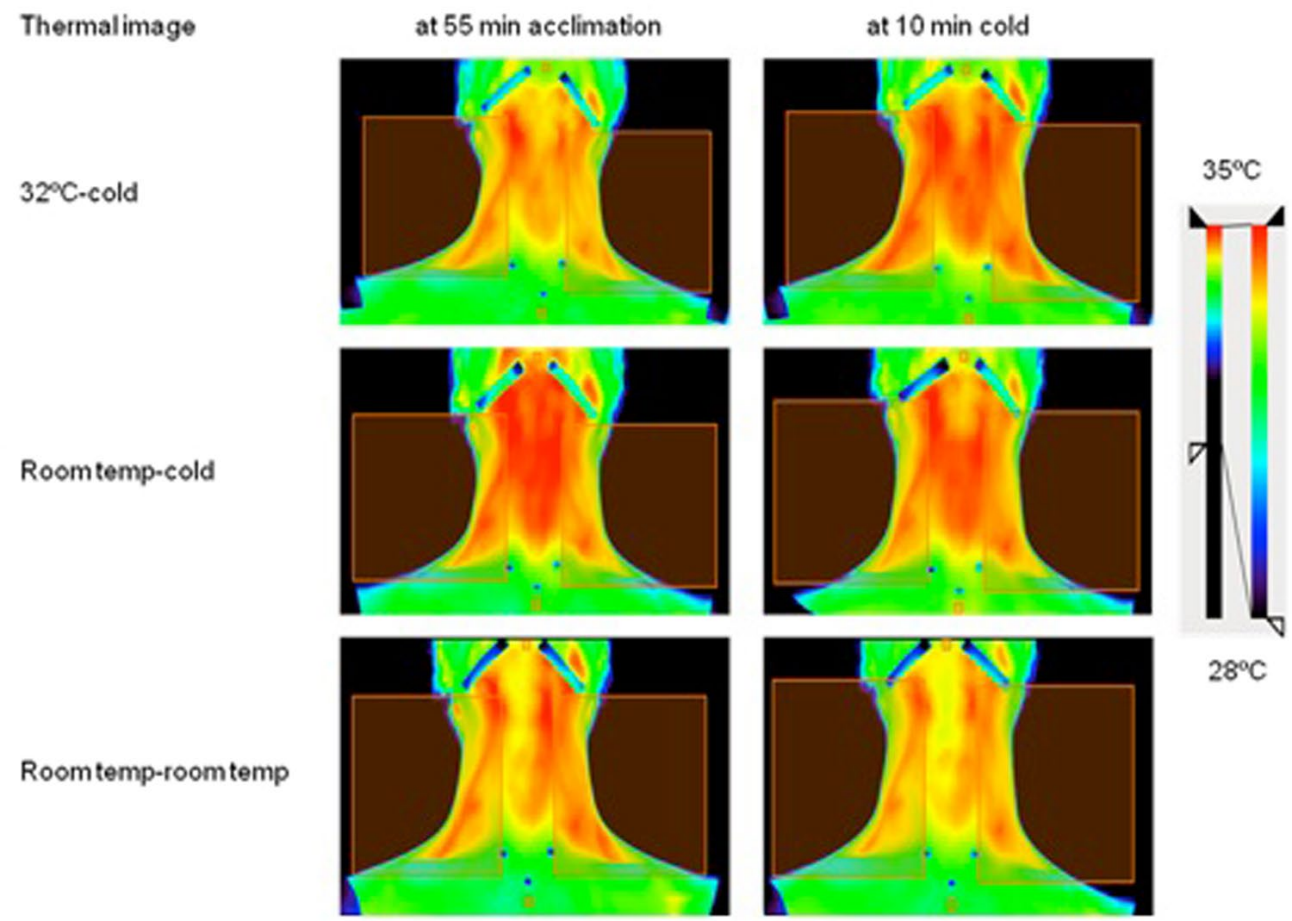
Thermal images of supraclavicular and cervical area showing ROIs before and after cooling in 3 trials in a participant with REE increase >100 kcal/d after cold exposure. Thermal images indicate that supraclavicular temperature is higher after cooling.

Participants changed into a light undershirt and sat fully upright in a chair placed one meter away from the camera with their head against a headrest (so head position remained constant). Thermal images were taken with an infrared camera (T650sc, emissivity of 0.98, FLIR Systems). Images were taken in the MSX (Multi Spectral Dynamic Imaging) mode. The images were analyzed with Amide software (data was formatted as float, little endian 32-bit, 0 offset bytes, with file size 1228800 bytes, dimensions 480 × 640 × 1, gates, frames and voxel size 1, scale factor 1000). They were aligned and overlapped before drawing the region of interest (ROI) which included right and left SCV areas. Anatomical landmarks were used to consistently define the area expected to contain BAT. These were rectangles delineated by the mandible superiorly, clavicle inferiorly, acromion laterally and sternoclavicular joint medially. Because the exact BAT location cannot be reliably predicted by external anatomy, the top 10 percent temperature of the larger, anatomically defined area was used (Fig. 2)^20^.

#### BAT activity

**Figure 3:**
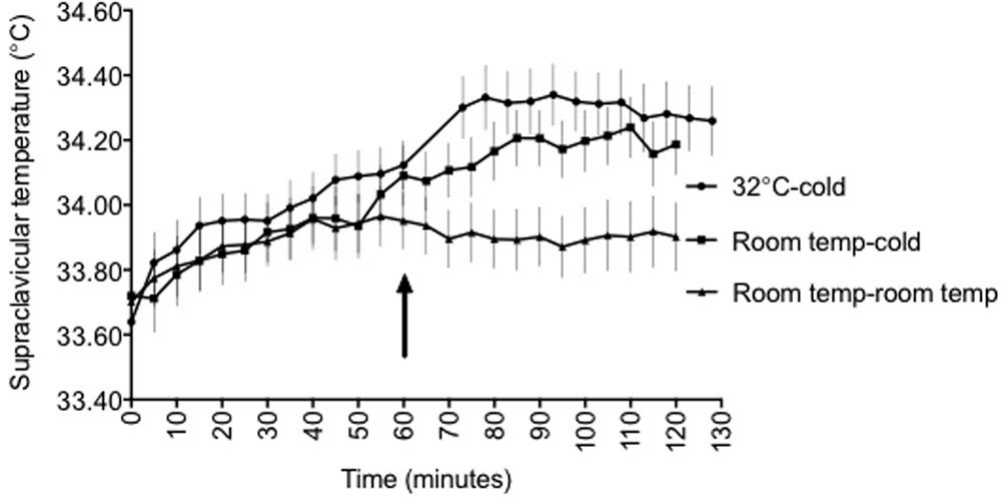
Supraclavicular temperature over time in 3 separate trials (n = 28). The arrow indicates when the acclimation period ended. Data are mean ±SEM.

The thermal response to cold was quantified by subtracting the baseline SCV temperature in the last 10 min of acclimation from the final temperature (delta) (Fig. 3). The SCV temperature over the last 10 min of 32 °C acclimation was steady and was therefore used as the baseline temperature prior to cold exposure.

#### Body composition measurements

Body composition was measured in Phase 1 with a PRODIGY DEXA scanner. Participants were instructed to change into a gown and gently lie on the scanner. The percent body fat was calculated with PRODIGY software after defining the ROIs. We delineated a ROI for the SCV and cervical region (neck fat), defined by a trapezoid area bounded by a line drawn just below the chin superiorly, a line joining the acromion processes inferiorly and lines connecting them on either side.

#### EMG

Eight surface fixed preamp electrodes (Motion Labs Inc.) were placed over the bilateral trapezius, pectoralis major, latissimus dorsi and quadriceps. The signal was then amplified and band-pass filtered (10–1000 Hz) using a 16-Channel EMG system (Motion Labs Inc.). Data processing was conducted using custom software designed on a Labview platform (National Instruments). Surface EMG was recorded for 1 min at a sampling rate of 1KHz at time 0 and then every 10 min over the entire hour. The raw EMG was qualitatively examined for burst activity indicative of shivering. A shivering burst was characterized according to a study by Haman at el who defined it by an EMG interval of >0.2 sec duration with interburst interval of >0.75 sec and amplitude higher than the amplitude threshold^25^.

Height was measured with a wall-mounted Harpenden stadiometre and weight with an electronic platform scale. Local outdoor temperature on the morning of the visit, and average temperature for the 2 months prior to the visit were recorded from the website http://climate.weather.gc.ca. Indoor temperature which was between 20–23 °C was measured with a digital room thermometer (Instant transmission plus).

### Statistical analysis

Values were expressed as mean ±SEM in the figures. Repeated measure ANOVA was used to compare the thermal response between the 3 trials in Phase 1 (32°-cold, room temp-cold and room temp - room temp trial). The univariate relationship of thermal response with neck fat and outdoor temperature was evaluated using Pearson’s correlation coefficient. Repeatability was calculated using intraclass correlation coefficient (ICC) (one-way, fixed model, average measures, consistency) with 95% confidence interval (CI). Independent student’s t test was used to determine any difference in the characteristics between participants with and without 100 kcal/d increase in REE. All statistical analysis was carried out with SPSS version 20.

## Results

### Phase 1: determination of the pattern and magnitude of thermal response

Of the 62 male participants tested, 28 (mean age 24.0 ± 5.9 years) had a 100 kcal/d increase in REE with cold exposure and underwent thermography at subsequent visits. Burst activity indicative of high intensity shivering on EMG was not seen in any of the muscles throughout the 1 hour period of 12 °C cooling. This excluded the presence of high intensity shivering, and confirmed that the increase in energy expenditure at 12 °C was likely the result of nonshivering thermogenesis. The participants were of Caucasian (18), Asian (9) and African (1) origin. Mean BMI was 25.2 ± 3.9 kg/m^2^, mean lean body mass was 58.6 ± 6.2 kg and mean percent body fat was 19.6 ± 8.0%. Clinical characteristics are shown in Table 2.

**Table 2:** Description of participants in Phase 1 who had a 100 kcal/d increase in REE after cooling.

Variables	n	mean ± SD
Age (years)	28	24.0 ± 5.9
BMI (kg/m^2^)	28	25.2 ± 3.9
Lean body mass (kg)	25	58.6 ± 6.2
Body fat percentage (%)	25	19.6 ± 8.0
Neck fat percentage (%)	25	9.5 ± 5.4
Outdoor temperature in (°C)		
- indirect calorimetry trial	28	7.6 ± 12.3
−32 °C-cold trial	28	9.6 ± 12.5
- room temp-cold trial	28	11.3 ± 12.4
- room temp-room temp trial	28	11.3 ± 11.9
-average 2 months prior to 32 °C-cold trial	28	4.8 ± 10.6
Baseline REE (kcal/d)	28	1490.7 ± 234.2
Cold induced REE (kcal/d)	28	1719.9 ± 240.7
Change in cold induced REE (kcal/d)	28	229.3 ± 146.6

Note: DEXA scan could not be performed in 3 participants due to weight >116 kg and participant’s busy schedule.

#### Pattern and magnitude of thermal response

The SCV temperature increased with cold exposure both after 32 °C and room temperature acclimation, but remained steady in the room temp - room temp trial. The SCV temperature rose abruptly with cooling, plateauing at 10 min after 32 °C acclimation (Fig. 3).

**Figure 4:**
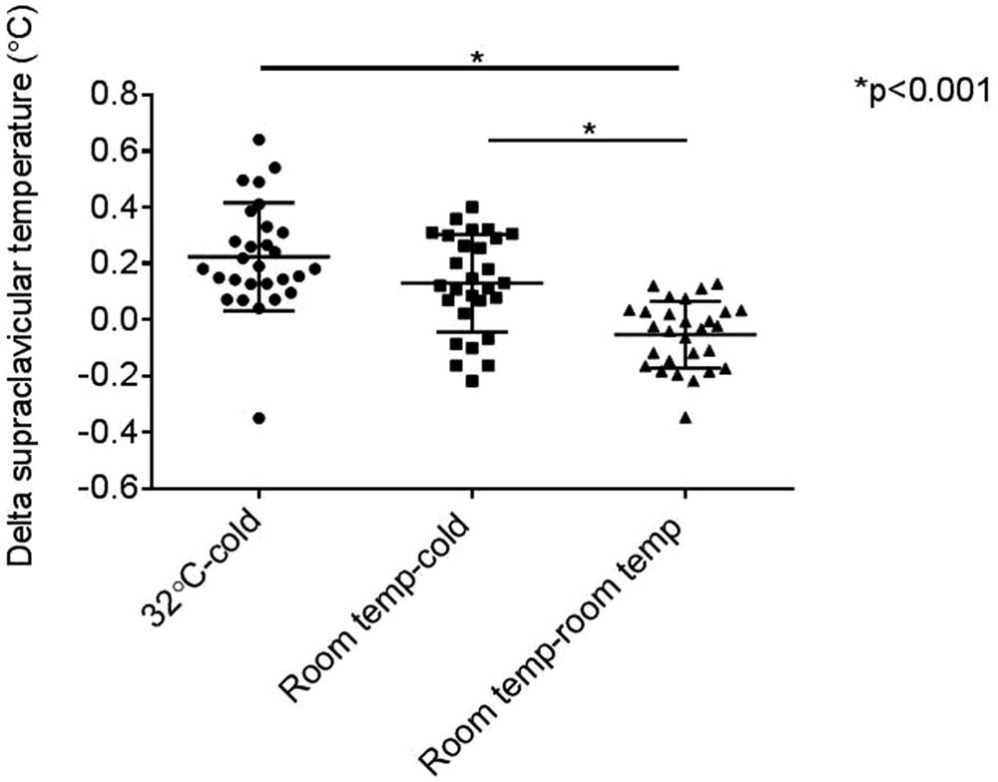
Comparison of thermal response in the 3 trials (n = 28). Thermal response was measured by delta supraclavicular temperature (Δ SCV temp), which is the average SCV temp between 10–25 min cold exposure less the average SCV temp over the last 10 min of acclimation (baseline). Data are mean ±SD.

The thermal response in the 32 °C-cold trial tended to be greater than that of the room temp-cold trial (0.22 ± 0.19 vs 0.13 ± 0.17, p = 0.053). Cold exposure increased SCV temperature in subjects acclimated to either 32 °C or room temperature compared to no cold exposure (0.22 ± 0.19 vs −0.05 ± 0.12, p < 0.001; 0.13 ± 0.17 vs −0.05 ± 0.12, p < 0.001) (Fig. 4).

#### Relation of thermal response with outdoor temperature

There was no correlation between outdoor temperature and thermal response in the 32 °C-cold trial (r = −0.35, p = 0.07) and room temp-cold trial (r = −0.07, p = 0.71) in 28 participants with 100 kcal/d increase in REE. There was no correlation between the average outdoor temperature in the 2 month period prior to the 32 °C-cold trial and the thermal response (r = 0.13; p = 0.52).

#### Relation of thermal response with neck fat

There was no significant correlation between percent fat in the cervical and SCV area and delta SCV temperature in the 32 °C-cold trial in 25 young males with BMI ranging from 19.3–32.3 kg/m^2^ (r = −0.26, p = 0.21).

Since a greater and earlier response to cold was seen in the 32 °C acclimation trial, we chose that as the acclimation temperature to utilize for the repeatability experiments in Phase 2.

### Phase 2: determination of the repeatability of SCV thermal response

Table 3 describes the study participants in Phase 2. Out of 14 participants in Phase 2, 7 showed a 100 kcal/d increase in cold induced REE. There was no difference in age, BMI, percent body fat or outdoor temperature between subjects with or without an increase in REE. Participants had a mean age of 20.9 ± 2.4 years and were relatively lean (mean BMI 23.6 ± 3.2 kg/m^2^).

**Table 3:** Description of male participants and mean (SD) REE and SCV temperature in Phase 2.

Variable	Participants with 100 kcal/d increase in REE		Participants without 100 kcal/d increase in REE		All participants	
	n	Mean ± SD	n	Mean ± SD	n	Mean ± SD
Age (years)	7	21.1 ± 2.8	7	20.7 ± 2.1	14	20.9 ± 2.4
BMI (kg/m^2^)	7	22.8 ± 2.8	7	24.4 ± 3.5	14	23.6 ± 3.2
**Outdoor temperature in (°C)**						
-indirect calorimetry trial	6	8.1 ± 8.8	7	6.9 ± 4.1	13	7.5 ± 6.4
−32 °C-cold trial 1	7	8.9 ± 9.5	7	10.5 ± 8.0	14	9.7 ± 8.5
−32 °C-cold trial 2	7	6.5 ± 6.1	7	6.0 ± 3.7	14	6.3 ± 4.8
−32 °C-cold trial 3	7	7.6 ± 7.1	7	8.0 ± 8.0	14	7.9 ± 7.3
-room temp-room temp trial	7	5.4 ± 10.3	7	4.5 ± 5.8	14	4.4 ± 7.9
Baseline REE (kcal/d)	6	1558.8 ± 179.1	7	1654.3 ± 139.7	13	1610.2 ± 159.9
Cold induced REE (kcal/d)	6	1782.9 ± 170.3	7	1618.3 ± 153.8	13	1694.2 ± 176.7
Change in cold induced REE (kcal/d)	6	224.1 ± 110.8	7	-36.1 ± 80.9	13	84 ± 163.1
**Delta SCV temperature (°C)**						
- trial 1	7	0.34 ± 0.20	7	0.18 ± 0.20	14	0.26 ± 0.21
- trial 2	7	0.31 ± 0.15	7	0.29 ± 0.24	14	0.30 ± 0.19
- trial 3	7	0.26 ± 0.17	7	0.23 ± 0.17	14	0.24 ± 0.16

Note: Participants were of Caucasian, Asian and African origin.

Indirect calorimetry had already been performed in Phase 1 in 1 participant.

#### Repeatability of thermal response

**Figure 5:**
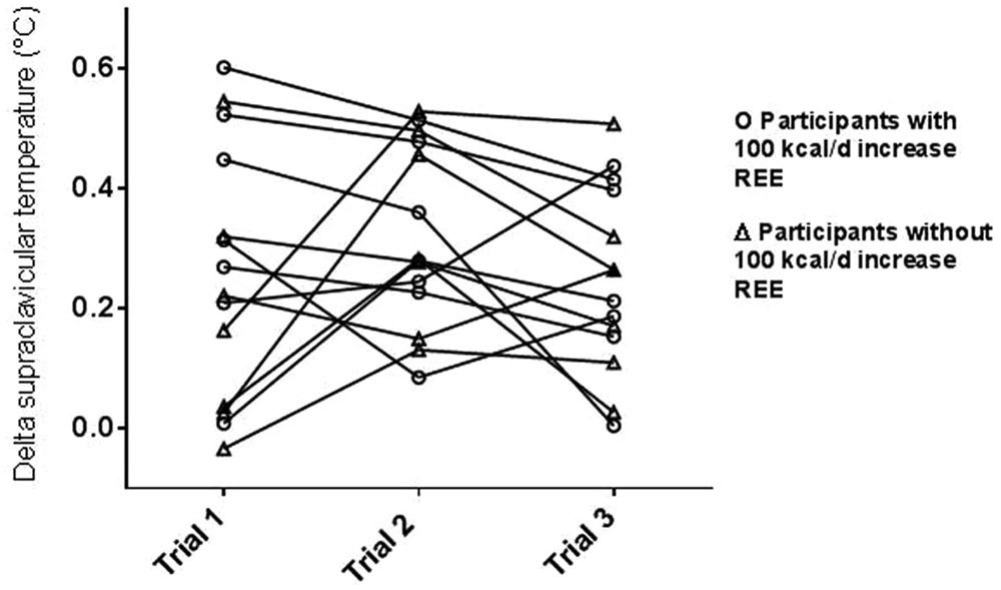
Repeatability of SCV thermal response in 3 × 32 °C – Cold Trials

Three 32 °C-cold thermography visits were performed approximately 9 (5–19) days apart with a mean difference of 5.7 ± 3.5 °C in outdoor temperature between the visits. The thermal response of each participant in the 3 trials is shown in Fig. 5. The ICC with 95% CI of delta SCV temperature in the 32 °C-cold trial was 0.69 (0.14–0.72).

## Discussion

Three previous studies have investigated the effects of cold exposure on SCV temperature in humans^20–22^. However, standardization of the cold exposure duration and the repeatability of the measure were uncertain.

In this study, we compared 2 different acclimation temperatures – RT and thermoneutral (32 °C). We observed that the response after the 32 °C-cold trial (i) is maximal after 10 min of cold exposure, (ii) results in a greater thermal response in the SCV area than room temp-cold or room temp-room temp response, (iii) is not influenced by outdoor temperature on the day of the test, (iv) is not related to regional neck fat in young healthy adult men with BMI ranging from 19.3–32.3 kg/m^2^ and (v) is highly reproducible.

In the current study, SCV skin temperature rose approximately 0.22 °C higher in the in the cold exposure trials compared to the 2 hour room temperature trial. Other studies have also shown a significant increase in SCV temperature upon cold exposure (0.20 ± 0.08 °C; 35.2 ± 0.1 to 35.5 ± 0.1 °C)^20,21^. Given the ability of BAT to generate heat, the thermogenic response which occurs upon exposure to cold most likely is indicative of BAT activity.

The skin temperature response was rapid, occuring within 10 min of cooling in the 32 °C acclimation trial. Symonds *et al*. also reported a rapid thermal response within 5 min of cold exposure only after a few min of acclimation at room temperature^20^. This is consistent with the rapid action of the sympathetic nervous system to increase BAT activity^1,20^. It is interesting that there was no further increase in the thermal response after it peaked at 25 min; instead it decreased even in the presence of ongoing cold exposure in the 32 °C-cold trial. This may be due to a homeostatic mechanism whereby thermal receptors have adapted to the prolonged stimulus. Generally, it is the property of all sensory mechanoreceptors to discharge at a high rate at the beginning of a constant stimulus. The response then progressively decreases until it finally stops^26^.

The thermal response in the 32 °C-cold trial was also of greater magnitude than in the room temp-cold trial. The SCV temperature became steady after 45 min of 32 °C acclimation, probably as temperature regulation occurs without any metabolic heat production at thermoneutrality, completely turning off BAT activity^27^. On the other hand, the SCV temperature gradually increased over the last 15 min of room temperature acclimation. This is in accordance with Symonds who showed that mild cooling at 19–20 °C stimulates BAT20. When individuals are exposed to 12°C cold from 32 °C, the abrupt temperature change probably triggers greater sympathetic activity. However at room temperature, there may be low level BAT thermogenesis contributing to the SCV temperature. As such, the change in temperature in the SCV region after introducing a 12 °C cold stimulus would not be as robust. Boon *et al*. also demonstrated an increase of similar magnitude (0.3 °C) after 1 h acclimation at 32 °C^21^. However, Jang *et al*. did not see an increase in SCV temperature on cooling (32.8 ± 0.3 to 32.3 ± 0.3 °C on the left and 32.4 ± 0.3 to 31.6 ± 0.3 °C on the right side) as BAT may already have been active because subjects were not acclimated to thermoneutral conditions before cooling^22^.

Environmental temperature and season may affect the prevalence of non-cold stimulated BAT activity^1,2,9,13,28^. Therefore we looked at the effect of outdoor temperature on the morning of the scan on thermal response, but found no correlation between them in the 32°C-cold or room temp-cold trials. This is in contrast with other studies that show an inverse relation with outdoor temperature^1,9,13,28^. However, they are all retrospective studies that measured unstimulated BAT (no cold-exposure) without an acclimation phase. In the current study, thermal response was measured after an hour of acclimation (either 32 °C) in an attempt to minimize the effect of external environmental temperature on BAT. The lack of relation with outdoor temperature seems more plausible after 32 °C acclimation, as facilitative thermogenesis shuts down at thermoneutrality^27^.

A limitation of the thermography technique is that a blunted increase in surface temperature on cold exposure could be due to increased neck fat and not reflective of reduced BAT activity. To address this concern we assessed the relationship between percent fat in the cervical and SCV area and thermal response. While we acknowledge that there is not a validated method to measure neck fat available, we chose to measure it using DEXA with appropriate anatomical markers (described above). No relation was found between neck fat and thermal response in 25 young males with BMI ranging from 19.3–32.3 kg/m^2^. This suggests that regional neck fat is not related to change in SCV temperature. This is consistent with the findings of Robinson *et al*. who noted that a high BMI was not a major factor affecting temperature over the SCV area^29^. We acknowledge however that DXA does not directly measure subcutaneous fat. Gatidis *et al*. reported that SCV skin surface temperature was inversely related to local subcutaneous adipose tissue thickness measured by CT scan at 21 °C in 102 subjects with mean BMI of 26 ± 5 kg/m^2^ (r = −0.65, p < 0.01)^30^. However, this relation was seen at a single time point in the absence of cold exposure. Although CT measurement of SCV subcutaneous fat would more definitely evaluate the influence of SC fat on cold-stimulated SCV temperature change, this has not yet been evaluated.

We determined the repeatability of the 32 °C-cold trial. The thermal response to the 32 °C-cold trial was highly reproducible. This conclusion is strengthened as the thermal response in participants with less than 100 kcal/d increase in cold induced REE was also reproducible.

A limitation of the study is that the thermal response was not validated against other measures of BAT volume or activity. Thermography has however, been validated against 18F-FDG PET-CT by others. A key next step in this method development would be validate against other measures of BAT activity including MRI. Since the 3 trials in phase 1 were not randomized or blinded, the participant’s anticipation of cold may have affected their sympathetic activity. We were also not able to perform a deep muscle EMG with concurrent indirect calorimetry or calculate low intensity shivering in terms of maximum voluntary contraction.

Lastly, we only studied young healthy male participants; therefore the study should be repeated on a larger scale and in a more heterogeneous population to confirm the validity of results in other populations.

In conclusion, these data suggest that acclimation at 32 °C followed by 12 °C cold generates a significant and reproducible change in SCV skin temperature that may be indicative of BAT metabolic activity in adult humans. Furthermore, BAT detection with the 32 °C-cold protocol using infrared thermography is unlikely to be affected by environmental temperature and subcutaneous neck fat. Safe measurement of BAT in humans will aid the investigation of this novel tissue for the treatment of metabolic disorders.
